# Persistence and Viability of *Lecanicillium lecanii* in Chinese Agricultural Soil

**DOI:** 10.1371/journal.pone.0138337

**Published:** 2015-09-16

**Authors:** Ming Xie, Yan-Jun Zhang, De-Liang Peng, Jie Zhou, Xiao-Lin Zhang, Zhao-Rong Zhang, Jin-Jin Zhao, Yu-Huan Wu

**Affiliations:** State Key Laboratory for Biology of Plant Diseases and Insect Pests, Institute of Plant Protection, Chinese Academy of Agricultural Sciences, Beijing, the People’s Republic of China; IPK, GERMANY

## Abstract

The entomopathogenic fungus *L*. *lecanii* has been developed as biopesticides and used widely for biological control of several insects in agricultural practice. Due to the lack of isolation/count methods for *L*. *lecanii* in soil, the persistence of this fungus in soil appears to have attracted no attention. A selective medium and count method for *L*. *lecanii* in soil based on cetyl trimethyl ammonium bromide (CTAB) was developed, and then the persistence and viability of this fungus in soil were investigated under field conditions between 2012 and 2014. The results showed that the rate of recovery for *L*. *lecanii* in soil on the selective CTAB medium was satisfactory. The minimum CFUs for *L*. *lecanii* on the selective medium (0.5 g/L CTAB) was about 10^2^ conidia/g soil. The *L*. *lecanii* density in soil declined quickly in the first month after inoculation with fungal conidia, kept stable for 6 to 10 months, and then decreased gradually until undetectable. *L*. *lecanii* could persist for at least 14 months in the agricultural soil of northern China. The colony growth, conidia yield and germination rate on plates, as well as the median lethal concentration or times (LC_50_ or LT_50_) to aphids, mycelium growth in aphids and sporulation on aphids of *L*. *lecanii* did not change significantly during the persistence in soil. In general, the count method developed here was a very useful tool for monitoring the dynamics of natural or introduced *L*. *lecanii* populations in soil, and the data on the persistence of *L*. *lecanii* in soil reported here were helpful for biological control and environmental risk assessment.

## Introduction

Entomopathogenic fungi (EPF) play a crucial role in the control of pest insect populations in nature [1–2], and have been considered as an important supplement or attractive alternative to chemical insecticides [3–4]. Several species of EPF have been formulated, registered and used as mycoinsecticides in the world [5]. However the mycoinsecticides often do not match the efficacy of cheaper chemical pesticides in the field application, which is the primary obstacle to their commercialization and large-scale application [6–8]. The inconsistent performance of biological control agents is often associated with an incomplete understanding of the ecological constraints of the biological system in which they are placed. Most biological control agents need a threshold population level and certain survival period for efficient pest control [9]. In addition, the persistence of introduced microorganisms in the environment is also crucial for the assessment of risks that may result from their applications [10]. Thus the assessment of the post-application persistence of a biological control agent is important with respect to its biocontrol potential and performance [11].

Many laboratory studies conducted with *Beauveria* spp., *Metarhizium* spp. and *Paecilomyces* spp. have shed light on the manner in which the persistence and long-term efficacy of fungal propagules are affected by different factors in the soil environment: by soil type [12–13], moisture conditions [14], temperature, pH, organic matter content and level of conductivity [15–16] and antagonistic organisms [17]. In the last two decades, several semi-field or field studies had been conducted with *Beauveria* spp. and *Metarhizium* spp [18–23]. These studies showed that the persistence time varied among EPF after the application, ranging from a few months to several years. The persistence depends largely on the environmental conditions of each area and the fungal isolate used. Hence, the extrapolation of results to other regions is not straightforward. In addition, the viability of EPF during the persistence in soil appears to have attracted no attention.

The entomopathogenic fungus *L*. *lecanii* is capable of infecting various pest insects, has a broad geographical distribution, and shows promise in commercial development [24]. However, there is no report about the persistence of *L*. *lecanii* in soil. Unlike *Beauveria* spp., *Metarhizium* spp. and *Paecilomyces* spp., an efficient selective medium for *L*. *lecanii* is absent, and the *Melolontha melolontha* bait method is also useless for *L*. *lecanii* because *M*. *melolontha* is not susceptible to *L*. *lecanii*. Although the molecular techniques based on PCR provide the necessary tools to undertake environmental sampling to monitor EPF (e.g. *B*. *bassiana* and *P*. *lilacinus*) [25–26], the PCR method is also unavailable to detect *L*. *lecanii* in soil due to the difficulty in obtaining pure DNA (unpublished).

In the present study, we developed an efficient selective medium for *L*. *lecanii*, and then investigated the persistence of this fungus in field soil in a 3-year experiment designed to see whether *L*. *lecanii* would have sufficient persistence in northern China for long-term protection of crops. At the meantime, the viability of recovered *L*. *lecanii* from soil was also studied. These results were important for us to understand the possible constraints of using EPF and to optimize its application strategies.

## Materials and Methods

### Ethics statement

The study presented no ethical issue.

### Fungal isolates

Nine isolates representing nine fungal species (*L*. *lecanii*, *B*. *bassiana*, *M*. *anisopliae*, *P*. *lilacinus*, *Verticillium dahlia*, *Aspergillus niger*, *Fusarium oxysporum*, *Alternaria alternate* and *Penicillium digitatum*) were investigated. Details on all fungal isolates are provided in Table 1.

**Table 1:** Origin of fungal isolates used in tests to evaluate the effectiveness of CTAB.

Isolates	Isolation source	Locality
***L*. *lecanii***		
FZ9906	Tea garden soil	Fuzhou, Fujian, China
KM9605	Forest soil	Kunming,Yunnan, China
LF1006	*A*. *gossypii* (Homoptera:Aphididae)	Langfang, Hebei, China
DZ1107	Tomato field soil	Dezhou, Shandong, China
GZ0306	*Ceroplastes rubens* (Hemiptera:Coccidae)	Ganzhou, Jiangxi, China
YQ1105	Cucumber field soil	Yanqing, Beijing, China
CZ1009	Tomato field soil	Cangzhou, Hebei, China
HS9310	Tea garden soil	Huangshan, Anhui, China
**Non-target fungi**		
*A*. *alternate*	Tomato field soil	Langfang, Hebei, China
*A*. *niger*	Cucumber field soil	Langfang, Hebei, China
*B*. *bassiana*	Forest soil	Wuyi, Fujian, China
*F*. *oxysporum*	Cucumber field soil	Langfang, Hebei, China
*M*. *anisopliae*	Tomato field soil	Langfang, Hebei, China
*P*. *lilacinus*	Soybean field soil	Langfang, Hebei, China
*P*. *digitatum*	Cucumber field soil	Langfang, Hebei, China
*V*. *dahlia*	Cotton field soil	Urumqi, Xinjiang, China

### Development of a selective medium for counting *L*. *lecanii* in soil

Oatmeal agar (OA) was used as the basal medium, which consists of 20 g/L rolled oatmeal and 20 g/L agar, and amended with 0.5 g/L chloramphenicol to retard bacterial growth. Three concentrations of CTAB (Amresco, Ohio, USA), 0.4 g/L, 0.5 g/L and 0.6 g/L respectively, were added to the basal medium to develop the testing medium. For comparison, OA with 0.5 g/L dodine (OA-D) (MingDou Chemical, Shandong, China) was prepared according to Chase et al [27].

Conidia of each fungus were collected by scraping the surface of PDA plates 10 days after incubation with the end of a sterile micropipette tip, and then suspended in 2 mL of sterile 0.05% (v/v) Tween 80 water and vortexed well. Each conidial suspension was filtered through four layers of Whatman lens cleaning tissue to eliminate mycelium, and then adjusted to 10^5^ conidia/mL with an improved Neubauer chamber (Hausser Scientific, PA, USA).

Conidial suspensions were diluted 1/10^2^. 100 μL aliquots of each diluted conidial suspension were spread with sterile glass spatulas uniformly over the surface of the testing plates. The control was performed on OA plates without CTAB. Four replicates were performed for each treatment. All plates were incubated at 25°C for 5 days. Plates with 15–150 colonies, the maximum number that can be distinguished with accuracy, were selected and the number of colonies were counted and expressed as colony forming units (CFU). For each treatment, the rate of recovery was calculated using the equations: the rate of recovery = (number of CFU on testing plates/number of CFU on OA plates) ×100%.

To test the intra-species variability on the selective CTAB medium, five different *L*. *lecanii* isolates were analyzed. 100 μL aliquots of each diluted conidial suspension (10^3^ conidia/mL) were spread on the OA medium containing 0.5 g/L CTAB supplemented with 0.5 g/L of chloramphenicol (OA-CTAB5). Four replicates were performed for each treatment. Plates were incubated at 25°C for 5 days. The number of colonies developed were counted and expressed as CFU. For each treatment the rate of recovery was calculated in the same manner as the above assay.

The minimum *L*. *lecanii* CFUs needed to develop on selective media after soil dilutions was also assayed. The natural soils (1 kg) were collected to a depth of 20 cm with sterile metallic spoons from Langfang in Hebei province of China. Conidial suspensions (10^5^ conidia/mL) were added into non-sterile or sterile soils, with final conidial concentrations of 10^5^ conidia/g, 10^4^ conidia/g, 10^3^ conidia/g, 10^2^ conidia/g, and 10^1^ conidia/g respectively. Sterile water was added into non-sterile or sterile soils as the control. One gram treated soils were diluted in 10 mL sterile Tween 80 (0.05%), and 100 μL aliquots of soil suspension were spread on the OA medium containing 0.5 g/L CTAB supplemented with 0.5 g/L of chloramphenicol. Four replicates were performed for each treatment. Plates were incubated at 25°C for 5 days. The number of colonies developed were counted and expressed as CFU.

### Production of the fungal inoculum

*L*. *lecanii* isolate FZ9906 was maintained on PDA slants and grown at 25°C for 10 days. Fungal conidia were harvested by washing slants with sterile 0.05% (v/v) Tween 80 water. The resulting conidia suspension was vortexed well, and adjusted to 10^7^ conidia/mL with an improved Neubauer chamber (Hausser Scientific, PA, USA). The conidia suspension was used as the first seed culture to inoculate the subsequent liquid-state fermentation immediately. Erlenmeyer flasks containing liquid media (yeast extract 20 g, glucose 25 g in 1 liter of water) were inoculated with the conidia suspension, and then fermented at 25°C, 200 rpm for 2 days. For the solid-state fermentation, the above fermented mixture was used as the second seed culture to inoculate. Rice (250 g) was mixed with 60 mL tap water and 4 mL soybean oil in a PE plastics bags and autoclaved at 121°C for 30 min. After cooling down to room temperature, each bag was inoculated with 50 mL second seed culture (about 10^7^ conidia/mL), and fermented at 25°C for 14 days in an incubator (Tayasaf, Beijing, China). The final cultural samples were dried at 38°C for 48 hours. Dried cultural sample (10 g) was mixed with sterile 0.05% (v/v) Tween 80 water and shaken for 10 min to separate spores from cultures. Conidia amount per gram cultural sample was calculated by counting the conidia suspension with an improved Neubauer chamber (Hausser Scientific, PA, USA). For the assessment of conidia germination, 50 μL of conidial suspension (10^5^ conidia/mL) were equally plated on water agar plates. The conidial germination rate was recorded after 16 h incubation at 25°C.

### Field design

**Fig 1:**
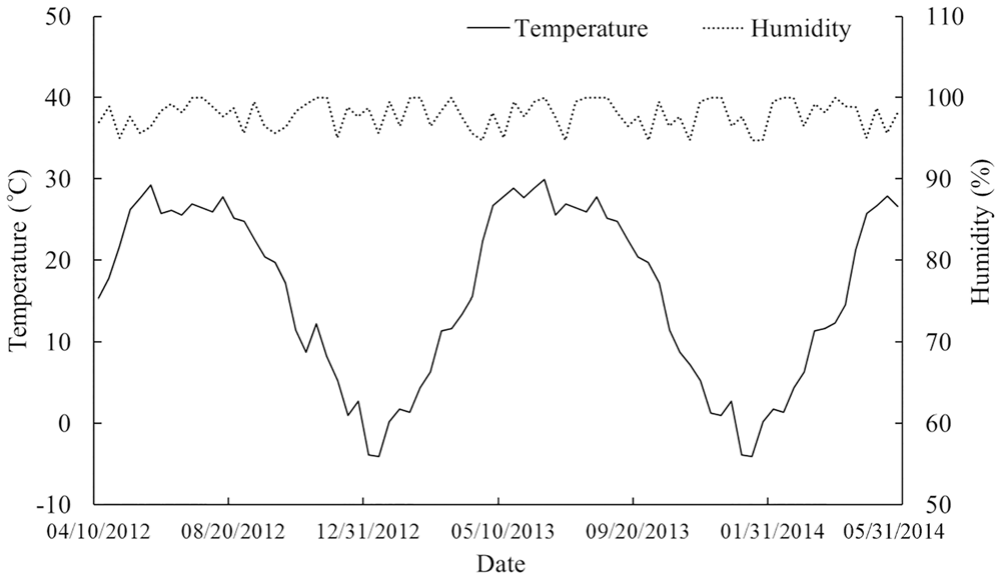
Fluctuation of mean dekad temperature and humidity in the soil during the trial.

Field trials were conducted at the tomato field on the experimental farm of the Institute of Plant Protection of the Chinese Academy of Agricultural Sciences, located at Cuizhuang town (39° 30′ N and 116° 36′ E,), Langfang, Hebei Province, China, during 2013–2014. The field has been infested by aphids or whiteflies. These insects had been exclusively controlled with the chemical pesticide imidacloprid. The fungus *L*. *lecanii* had not been previously applied as a biological control agent. This area is located in the North China Plain, which is in the North Temperate Zone with a continental monsoon climate, with an annual mean temperature of 11.8°C and annual mean rainfall of 570.3 mm. According to the WRB Soil Taxonomy, the soils at field plot sites belong to cambisols. The soil profiles consist of humus horizon (about 20 cm depth) and cambic horizon. The soil of the field site has the following properties (on a dry mass basis): pH (soil:water ratio 1:2.5) 8.4, organic matter 15.6 g kg^-1^, organic C 9.0 g kg^-1^, total N 1.0 g kg^-1^, total P 0.96 g kg^-1^, total K19.8 mg kg^-1^, available N 65.9 mg kg^-1^, available P 10.25 mg kg^-1^, available K 177.2 mg kg^-1^. The temperature and humidity of soil were monitored by an automatic recorder (Huatu, Shenzhen, China), and the results are shown in Fig 1.

### Soil inoculation and sampling

Dried cultural samples of *L*. *lecanii* were ground for 5 min in an all-purpose grinder (Tianjin Traditional Chinese Medicine Machinery Factory, Tianjin, China), and then applied into the soil in plots on 10 April 2012 and 8 September 2012 with two concentrations, 10^7^ conidia/g soil (high) or 10^5^ conidia/g soil (low) respectively. The soils inoculated with or without rice powder (no fungal conidia) were chosen as two controls. Four plots were performed as four replicates for each treatment. For each fungal inoculation, soil samples were collected in 10-day intervals in the first two months and subsequently one-month intervals. Soils before the fungal inoculation were also collected as the base samples. The sampling process continued until 15 April 2014. Each plot was divided into 100 sub-plots of equal size (100 cm^2^ aprox.) and four were selected randomly for the sampling in each date. These selected areas were not considered for subsequent sampling. On each sampling date, four 50 g subsamples were collected to a depth of 20 cm with sterile metallic spoons from sub-plots, mixed as a composite sample, and put in sterile plastic bags for transfer to the laboratory. Each soil sample was stored at 4°C until analysis (typically 1–2 days). One part was used to determine the dry weight of soil after drying at 105°C for 18 h. The other part was processed for enumeration of colony-forming units.

### CFU quantification of *L*. *lecanii* in the soil

Ten gram of each soil sample was suspended in 100 mL sterile 0.05% (v/v) Tween 80 water, shaken for 30 min at 220 rpm, and then 10-fold serially diluted to achieve dilutions of 10^−2^ and 10^−3^. 100 μL aliquots of diluted samples were spread on the OA medium containing 0.5 g/L CTAB supplemented with 0.5 g/L of chloramphenicol and incubated at 25°C for 5 days. Four replicates were performed for each treatment. Developing colonies with general characteristics of the species *L*. *lecanii* (colony color, conidia size, conidia shape, etc.) were removed from the Petri dish to confirm their identity by sequencing internal transcription spacer ribosome DNA amplified by the fungal universal primer pair of ITS1F/ITS4R as described previously [28–29]. *L*. *lecanii* colonies were quantified to determine the number of CFU/g in dry soil.

### Biological characteristics of recovered *L*. *lecanii* isolates from soil

*L*. *lecanii* colonies were recovered from soil samples by the OA medium containing 0.5 g/L CTAB supplemented with 0.5 g/L of chloramphenicol. Conidia suspensions of the original isolate FZ9906 and recovered isolates of *L*. *lecanii* were prepared as described above. The colony growth, conidial yield and conidial germination rate of *L*. *lecanii* isolates on plates were determined as described by Zhang et al [30]. The virulence of the original isolate FZ9906 and recovered isolates of *L*. *lecanii* was assayed with adult cotton aphids (*Aphis gossypii* Glover) as described by Hall [31]. The insects were placed in a net, immersed for 10 s in conidial suspension of five different concentrations (10^4^, 10^5^, 10^6^, 10^7^ and 10^8^ conidia/mL) and laid on sterilized filter paper to draw off surplus suspension. Control insects were treated with 0.05% (v/v) Tween 80 only. Each treatment had four replicates with 200 insects per replicate. The mortalities were recorded every 12 hours and cadavers were transferred to moisturized filter paper to monitor the emergence of fungal hyphae. The median lethal concentration (LC_50_) and median survival time (LT_50_) of the treated insects were calculated. To determine sporulation *in vivo*, dead insects were collected, weighed and maintained at 25°C and high humidity for 7 days. The sporulated dead insects were cut into very small pieces with a sterile knife and added to sterile water containing 0.05% Tween-80. The mixture was then stirred for 2 h before conidia were counted with an improved Neubauer chamber (Hausser Scientific, PA, USA). To determine the infection and extension *in vivo*, the growth of *L*. *lecanii* isolates in aphids was quantified by quantitative real-time PCR with the species-specific primer pair of *Lec*-F/R as described previously by Xie et al [32].

### Data analysis

Data were analyzed with the analysis of variance (ANOVA) and means were separated with the Fisher’s protected least significant difference (LSD) test at the 5% significance level to determine whether there were significant differences between treatments. All statistical analyses were performed with SPSS 19.0 (SPSS Inc., Chicago, USA).

## Results

### Assessment of the selective CTAB medium for *L*. *lecanii*

**Fig 2:**
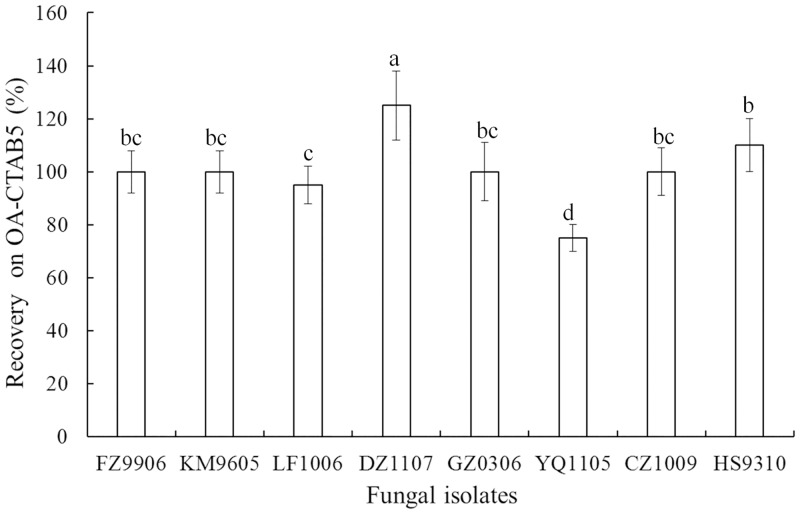
Intra-species variability of *L*. *lecanii* isolates on the selective medium (oatmeal CTAB 0.5 g/L). Letters above bars indicated statistical significance (LSD, P<0.05).

The CFU values of the fungal isolates on the culture media tested are provided in Table 2. The CFU values of *L*. *lecanii* showed no differences between the treatments and the control (P>0.05); the rates of recovery were always 100%. For non-target fungi, the CFU values of *B*. *bassiana* also showed no differences between the treatments and the control (P>0.05); the rates of recovery were always 100%. The CFU values of *M*. *anisopliae* on OA-D were significant with regard to the other treatments and the rate of recovery on OA-CTAB ranged from 48.5%-57.4%. The rate of recovery for *P*. *lilacinus* displayed significant differences between the control and the treatments (P<0.05). *A*. *niger*, *F*. *oxysporum* and *P*. *digitatum* did not grow on OA-D or any OA-CTAB media, whereas *A*. *alternate* and *V*. *dahlia* were detectable on OA-CTAB4 with the rate of 9.9% and 30.1% respectively. This study led us to select 0.5 g/L CTAB as the most effective concentration. All isolates of *L*. *lecanii* were able to grow on OA-CTAB5. Only the isolate YQ1105 was significantly different than the others (P<0.05) (Fig 2).

**Table 2:** Detection of different fungi on the culture media tested ^a^.

Fungal isolate	CFU values (Rate of recovery)				
	OA	OA-D	OA-CTAB4	OA- CTAB 5	OA- CTAB 6
***L*. *lecanii***	8.2×10^4^ a	8.5×10^4^ (100) a	9.8×10^4^ (100) a	9.8×10^4^ (100) a	8.9×10^4^ (100) a
***B*. *bassiana***	8.1×10^4^ a	9.8×10^4^ (100) a	9.5×10^4^ (100) a	8.7×10^4^ (100) a	8.2×10^4^ (100) a
***M*. *anisopliae***	6.8×10^4^ a	0.5×10^4^ (7.4) c	3.9×10^4^ (57.4) b	3.3×10^4^ (48.5) b	3.4×10^4^ (50) b
***P*. *lilacinus***	6.6×10^4^ a	3.3×10^4^ (50) b	3.5×10^4^ (53) b	2.9×10^4^ (43.9) b	2.7×10^4^ (40.9) b
***A*. *alternate***	9.1×10^4^ a	0.3×10^4^ (3.3) b	0.9×10^4^ (9.9) b	0 (0) c	0 (0) c
***A*. *niger***	8.8×10^4^ a	0 (0) b	0 (0) b	0 (0) b	0 (0) b
***F*. *oxysporum***	7.4×10^4^ a	0 (0) b	0 (0) b	0 (0) b	0 (0) b
***P*. *digitatum***	7.5×10^4^ a	0 (0) b	0 (0) b	0 (0) b	0 (0) b
***V*. *dahlia***	8.3×10^4^ a	0 (0) c	2.5×10^4^ (30.1) b	0 (0) c	0 (0) c

^a^ Data followed by different lowercase letters in a line are significantly different (LSD, P<0.05). The starting number of CFUs was about 1.0×10^5^. The rate of recovery = (number of CFU on testing plates/number of CFU on OA plates) ×100%. OA (basic medium): oatmeal agar, OA-D: oatmeal dodine 0.5 g/L, OA-CTAB4: oatmeal CTAB 0.4 g/L, OA-CTAB5: oatmeal CTAB 0.5 g/L, and OA-CTAB6: oatmeal CTAB 0.6 g/L.

Whether the soil was sterilized or not, it did not affect the CFU values obtained from inoculated soils with *L*. *lecanii* on the selective medium (OA-CTAB5). The rate of recovery for *L*. *lecanii* in soils was always 100% at high inoculation (≥10^4^ conidia/g soil). However it was 50% at the level of 10^3^conidia/g soil and decreased to 0% at low inoculation (≤10^2^ conidia/g soil). So the minimum CFUs needed to develop on the selective medium (OA-CTAB5) for *L*. *lecanii* in soil was about 10^2^ conidia/g (Table 3).

**Table 3:** CFU values obtained from inoculated soils with *L*. *lecanii* conidia on the selective medium (OA-CTAB5) ^a^.

Soil	10^5^ conidia/g	10^4^ conidia/g	10^3^ conidia/g	10^2^ conidia/g	10^1^ conidia/g	CK
**Non-sterile**	9.7×10^4^ a	9.8×10^3^ b	4.9×10^2^ c	0 d	0 d	0 d
**Sterile**	9.9×10^4^ a	1.0×10^4^ b	4.8×10^2^ c	0 d	0 d	0 d

^a^ Data followed by different lowercase letters in a line are significantly different (LSD, P<0.05). OA-CTAB5: oatmeal CTAB 0.5 g/L.

### Persistence of *L*. *lecanii* in soil

**Fig 3:**
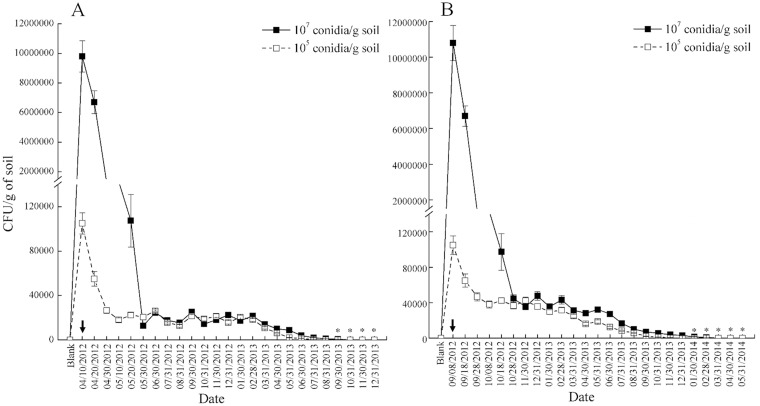
Fungus densities in the soil inoculated with *L*. *lecanii* conidia. Asterisk indicated that *L*. *lecanii* had not been detected from treated soils using the selective medium (oatmeal CTAB 0.5 g/L).

Two sampling analyses on 10 April 2012 and 8 September 2012 showed no CFU of *L*. *lecanii* in field soil before the inoculation of the fungal isolate FZ9906. After the first fungal inoculation (10 April 2012), the number of *L*. *lecanii* CFUs/g in soil were declining before the last soil samples were collected on 31 December 2013 (Fig 3A). The decline of the fungus was very rapid in the first 30 days or 50 days with the inoculation concentration of 10^7^ conidia/g soil (high) or 10^5^ conidia/g soil (low) respectively. From then on, the number of *L*. *lecanii* in soil kept stable and persisted at a low density (about 2×10^4^ CFUs/g) until 28 February 2013. Fungal densities continued to decline until they were undetectable in the soil from 31 August 2013 onward. A similar pattern of fungal persistence was observed for the second fungal inoculation (8 September 2012) (Fig 3B). Compared to the first fungal inoculation, we observed a significantly shorter stable period (about half) and significantly higher peak density (about two times) in the second inoculation.

### Viability of *L*. *lecanii* in soil

**Fig 4:**
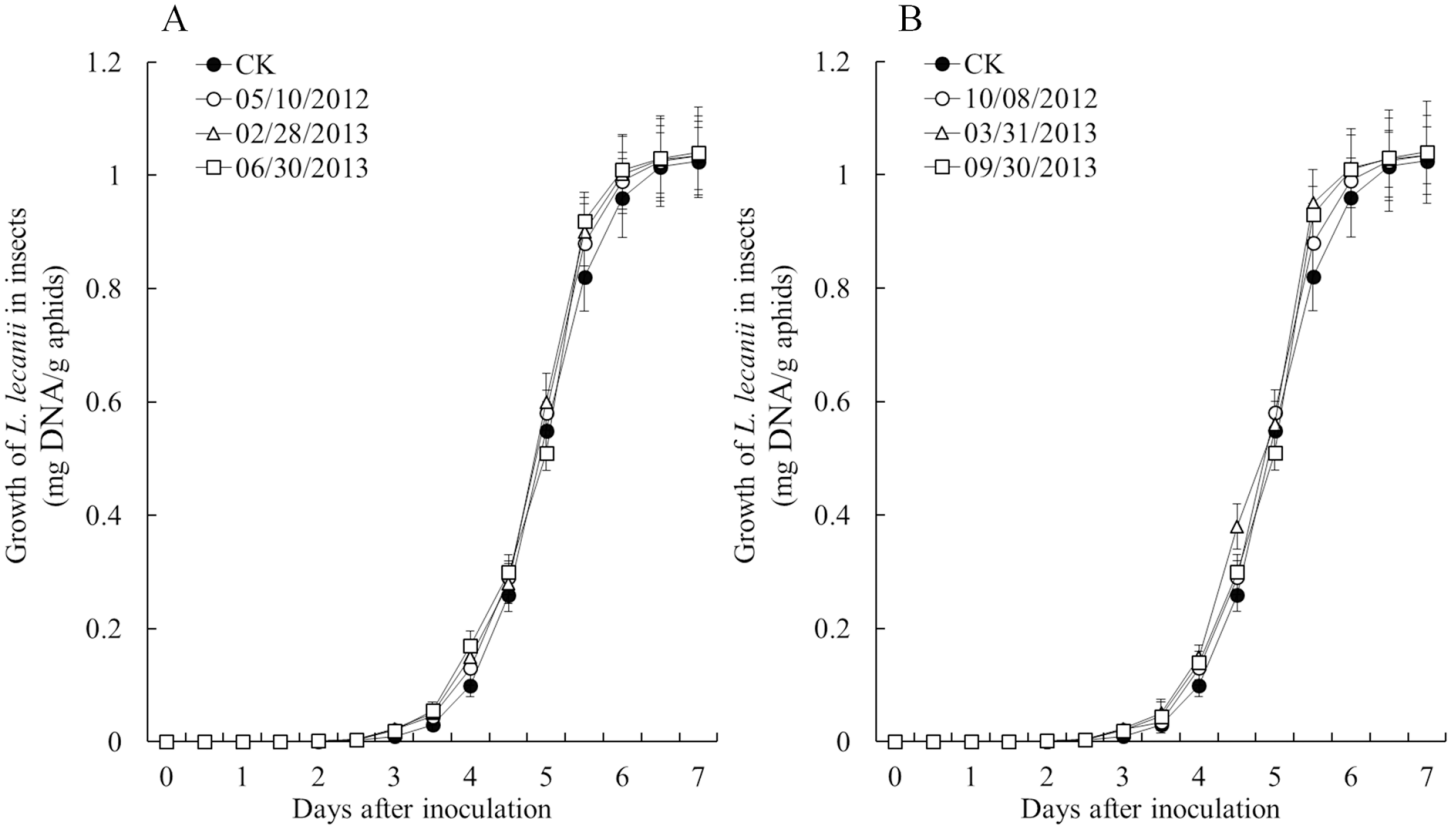
The growth of *L*. *lecanii* in cotton aphids measured using qPCR. Cotton aphids were inoculated with a concentration of 1×10^7^ conidia/mL by immersing.

Isolates recovered from the soils collected on three sampling dates for each fungal inoculation were analyzed and compared with the original isolate FZ9906. In the vitro assay of colony growth, conidia yield and germination rate, recovered isolates were not significantly different from the original isolate (P>0.05) (Table 4). In the vivo assay of median lethal concentration (LC_50_) to aphids and sporulation on dead cotton aphids, there were also not significantly different between the recovered isolates and the original isolate (P>0.05) (Table 4). Although the median lethal times (LT_50_) to aphids tended to decrease in the recovered isolates, a significant difference was not found between the recovered isolates and the original isolate. The results by qPCR also showed that there was no significant difference on the fungal growth of recovered isolates and the original isolate (P>0.05), although the growth of recovered isolates in aphids seemed to be slightly rapid (Fig 4).

**Table 4:** Comparison of biological characteristics of original and recovered isolates of *L*. *lecanii* ^a^.

Isolates	*In vitro*			*In vivo*		
	Colony growth (mm)	Conidial yield (10^6^ conidia/mm^2^)	Conidial germination (%)	LC_50_ to aphids (10^6^ conidia/mL)	LT_50_ to aphids (days) ^b^	Sporulation (10^9^ conidia/g) ^c^
**First inoculation**						
CK	22.5 a	2.3 a	98.0 a	1.6 a	3.5 a	3.5 a
05/10/2012	22.3 a	2.0 a	97.9 a	1.6 a	3.4 a	3.5 a
02/28/2013	22.2 a	2.2 a	98.0 a	1.6 a	3.3 a	3.4 a
06/30/2013	22.1 a	2.1 a	98.1 a	1.6 a	3.3 a	3.5 a
**Second inoculation**						
CK	21.9 a	2.4 a	97.8 a	1.6 a	3.4 a	3.3 a
10/08/2012	21.8 a	2.3 a	96.9 a	1.6 a	3.3 a	3.2 a
03/31/2013	22.2 a	2.1 a	97.9 a	1.6 a	3.2 a	3.3 a
09/30/2013	21.9 a	2.2 a	97.6 a	1.6 a	3.2 a	3.3 a

^a^ Data followed by different lowercase letters in a column are significantly different (LSD, P<0.05).

^b^ Aphids were inoculated with a concentration of 2×10^6^ conidia/mL by immersing.

^c^ Aphids were inoculated with a concentration of 1×10^7^ conidia/mL by immersing.

## Discussion

It is a very useful method to isolate/count EPF directly from the environment for monitoring the incidence and/or persistence of natural or introduced entomopathogenic fungal populations. Two of the most commonly employed methods are: (1) baiting the fungi from the environment with a susceptible insect host [33] or (2) using specific selective media containing chemicals that preclude or reduce the growth of contaminants [13–16,23,34–35]. Initially, the medium used for the isolation of EPF was Veen’s medium, which was aimed to culture general fungi and only semi-selective for EPF [34]. A variety of fungicides have been used in selective medium for EPF thereafter [35–36], such as benomyl, thiabendazole, and dodine. At certain concentrations, dodine effectively reduces the conidial germination and growth of most saprophytic and plant pathogenic fungi; while most EPF species tend to be less vulnerable to its effects [37–38]. These reports led to the development of a widely utilized dodine-based selective medium for isolation/count of EPF. Recently, dodine has experienced a sharp reduction in its primary market as a fungicide and thus is increasingly difficult to obtain [39]. Accordingly, dodine-free selective media were developed [35,40].

Our current study succeeded firstly in developing an effective selective medium for counting the entomopathogenic fungus *L*. *lecanii* in soil. The rate of recovery for *L*. *lecanii* was similar and always 100% on OA-D and OA-CTAB (Table 2). For non-target fungi, *A*. *niger*, *F*. *oxysporum*, *P*. *digitatum*, *A*. *alternate* and *V*. *dahlia* were completely inhibited by CTAB at 0.5 g/L, whereas *B*. *bassiana*, *M*. *anisopliae* and *P*. *lilacinus* still could grow on all CTAB concentrations. These was in agreement with the results by Posadas et al [40], who observed that CTAB almost did not affect the growth of *B*. *bassiana*, but significantly precluded the growth of *M*. *anisopliae* and *P*. *lilacinus*. However the colony of *L*. *lecanii* was easy to be identified from those of *B*. *bassiana*, *M*. *anisopliae* and *P*. *lilacinus* on the CTAB medium. All *L*. *lecanii* isolates displayed tolerance to CTAB in the present study regardless of the origin (Fig 2). Whether the soil was sterile or non- sterile, the minimum CFUs of *L*. *lecanii* in soil was as low as 10^2^ conidia/g on OA-CTAB5 (Table 3). Overall, the recovery of *L*. *lecanii* on this selective CTAB medium was up to expectation.

Determination of the fungal density based on the above count method revealed that the entomopathogenic fungus *L*. *lecanii* could persist in soil for at least 14 months in northern China. The persistence of *L*. *lecanii* was shorter than the 3-year period for *M*. *anisopliae* [19] and the 14-year period for *B*. *brongniartii* [20], however it was in the same range as 15-months for *M*. *anisopliae* [21] or 16-months for *M*. *acridum* [23]. As described in introduction, many factors could influence the persistence of EPF in the soil, e.g. soil type, weather conditions, and method of fungal inoculation, etc. In the present study, the high inoculation rate led to a less dramatic decline of *L*. *lecanii* CFUs at the beginning no matter the season of fungal inoculation was in spring (10 April 2012) or autumn (8 September 2012). The season of fungal inoculation also affected the persistence of *L*. *lecanii* in the soil. The stable period was significantly shorter (about half) and the number of *L*. *lecanii* CFUs was significantly higher (about two times) in spring than in autumn inoculation (Fig 2). On the contrary, the decline of *P*. *lilacinus* density was not significantly affected by inoculation rate [16]. The soil’s microorganism carrying capacity is limited, so the redundant or unadapted microorganisms have to die or degrade [41]. This may be the reason why the number of *L*. *lecanii* CFUs in soil gradually declined to an undetectable density after fourteen months. In addition, the recovery of *L*. *lecanii* from soils on the selective medium was not linear and 0% at low fungal inoculation (≤ 10^2^ conidia/g soil) (Table 3). This result indicated that the recovery of *L*. *lecanii* was interfered by the unknown soil factor like pH, soil organic content and composition etc. However the minimum CFUs of 10^2^ conidia/g soil on the selective medium could be considered useful for monitoring the density of *L*. *lecanii* in the field soil.

Another interest in the present study was to investigate the viability of *L*. *lecanii* after a period of persistence in soil. The mycelium growth and conidia yield on plates, and conidial germination rate of recovered isolates showed no difference from the original isolate. LC_50_ to aphids and sporulation on insects were also did not change. LT_50_ to aphids or fungal growth in insects seemed to decrease or increase in the recovered isolates, but no significant difference was found. As for the slight increase of virulence (shorter LT_50_ or more fungal expansion in hosts), it might due to the induction by insect hosts or starvation conditions in soil [42–43].

To conclude, an effective selective medium for counting the entomopathogenic fungus *L*. *lecanii* in soil was developed. Using this selective medium (0.5 g/L CTAB), we found that the entomopathogenic fungus *L*. *lecanii* could persist in agricultural soil for at least 14 months in northern China. The growth, sporulation, germination and aphid virulence of *L*. *lecanii* did not change significantly during its persistence in soil. Due to the complexity of the environments into which the fungus was applied, further research is warranted to investigate the ability of *L*. *lecanii* to persist and establish in the environment under various climates and across different field locations.
